# STOREFISH 2.0: a database on the reproductive strategies of teleost fishes

**DOI:** 10.1093/database/baaa095

**Published:** 2020-11-20

**Authors:** Stéphane Teletchea, Fabrice Teletchea

**Affiliations:** UFIP, Université de Nantes, UMR CRNS 6286, 2 rue de la Houssinière, 44322 Nantes cedex 3, France; University of Lorraine, INRAE, UR AFPA, 2 avenue de la Forêt de Haye - BP 20163 , F-54000, Vandoeuvre-lès-Nancy Cedex, France

## Abstract

Teleost fishes show the most outstanding reproductive diversity of all vertebrates. Yet to date, no one has been able to decisively explain this striking variability nor to perform large-scale phylogenetic analyses of reproductive modes. Here, we describe STrategies Of REproduction in FISH (STOREFISH) 2.0, an online database easing the sharing of an original data set on reproduction published in 2007, enriched with automated data extraction and presentation to display the knowledge acquired on temperate freshwater fish species. STOREFISH 2.0 contains the information for 80 freshwater fish species and 50 traits from the analysis of 1219 references. It is anticipated that this new database could be useful for freshwater biodiversity research, conservation, assessment and management.

**Database URL**: www.storefish.org

Teleost fishes are the most speciose taxa of vertebrates with >34 000 species described so far (1). They have colonized almost every possible marine and freshwater habitats from tropics to polar regions (2). They displayed the most outstanding reproductive diversity of all vertebrates (2–4). This includes, among others, egg diameter, larval size, gender systems, spawning dynamics, modes of fertilization, mating systems, secondary sexual characteristics and parental care (2, 3). Yet to date, no one has been able to decisively explain this striking diversity nor to perform large-scale phylogenetic analyses of reproductive modes (3, 4). This is mainly due to the lack of information on the reproduction of numerous species (3) despite the recent progresses on their phylogeny (∼80% of the families) (5). Therefore, only few studies attempted analysing the evolution of a handful of reproductive traits for a large number of species (6) while many more works compared numerous traits but for a low number of species (7). The comparative analysis of reproduction of teleosts is useful to understand trade-offs between reproductive traits (e.g. oocyte diameter and fecundity) and highlights common patterns of life history (e.g. 8, 9). Reproductive traits are also increasingly being used, often combined with other traits, in a wide range of applications in ecological and evolutionary research (10–12), such as for river (13) and fisheries (14) management, to predict fish invasions (15) or for the assessment of climate change (16).

Trait-based approaches first require gathering data on several traits, which could be defined as measurable ecological, life history, morphological, physiological and behavioural expressions of species’ adaptations to their environment (11). Cano-Barbacil *et al.* (12) recognized two types of traits (1): biological traits describing life cycle, physiological and/or behavioural characteristics including maximum body size, longevity, or feeding and reproductive strategies and (2) ecological traits or requirements that are linked to habitat preferences, water flow, pollution or temperature tolerances (12). This essential step is time-consuming and usually considered tedious and tricky to perform because no efficient automatic system exists (17–19). Despite progress to transform data described in natural language (free text) into a computable database that can then be statistically analysed, it is indeed still necessary to manually search information in each reference (18). This explains why phenomics—as a counterpart to genomics—which aims to make large-scale comparisons of phenotypes, is still in its infancy, and today only semi-automatic approaches appear feasible (20) [see, for instance, refs. (17, 21)]. For fish, the largest database ever developed is FishBase [www.fishbase.org (22, 23)]. Created by Daniel Pauly and Rainer Froese in the late 1980s (http://www.seaaroundus.org/tag/fishbase/), this database was initially conceived to service the fisheries science community and has since evolved to cover many aspects of the life history of fish (24). It is today the electronic encyclopaedia on fish (25) and in recent years has received more citations per year than any other fisheries reference (26). Many other databases were also developed for fish, such as FishTraits [http://www.fishtraits.info/ (11)], FishTEDB [http://www.fishtedb.org/ (27)], Osteobase [http://osteobase.mnhn.fr/ (28)], FishEthoBase [http://www.fishethobase.net/ (29)] or TOFF [http://toff-project.univ-lorraine.fr (30)], often with links to FishBase.

**Figure 1.**
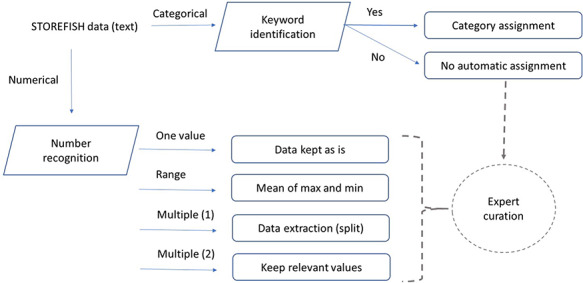
Data processing and feature extraction workflow. Traits defined as numbers were extracted using python’s regular expression, text entries were extracted semi-automatically by keywords extraction. Incomplete or difficult data extraction were curated by experts.

In 2005, a research programme was launched to evaluate whether it is possible to extrapolate the zootechnical knowledge acquired from one species to others to help diversifying aquaculture production (31). The rationale was that if clusters of species were sufficiently homogeneous, this could allow predictions of biological patterns between species (31). Once completed, this approach would allow the application of existing zootechnical technologies proven to work on one species to others belonging to the same cluster, thus lowering the uncertainty when farming a new species and saving both time and money (32–34). In order to evaluate the reliability of such a comparative approach, reproduction was used as a proof-of-concept function because its control is a prerequisite for domesticating new ﬁsh species (31). This research programme was restricted to temperate freshwater ﬁshes inhabiting chieﬂy Western Europe because European inland aquaculture had several opportunities for diversiﬁcation (31). We also anticipated that enough reliable information was available in the literature (31). Because FishBase did not contain enough data for the targeted species at the time, the first goal of this project was to develop a new database, which was entitled STOREFISH, acronym for STrategies Of REproduction in FISH (31). The entire development of the STOREFISH database was divided into four main tasks [see Figure 1 in Teletchea (35)]. The first task was to establish a structured and semantically formalized ontology (17, 19), which could be defined as a controlled vocabulary that describes objects and the relations between them in a formal way (36). Based on a 6-month literature search, a preliminary list of 135 traits was established, among which 50 were finally kept. For each of them, a definition was proposed and validated by a group of 10 fish specialists (31). Among the 50 biological and ecological traits (Table 1), 23 are categorical (e.g. egg buoyancy) and 27 are numerical (e.g. oocyte diameter) [see also Frimpong and Angermeier (11)]. They were grouped into five main categories: 7 traits for egg, 7 for larvae, 12 for females, 9 for males and 15 for spawning conditions (31). The second task was to select species among the 150 initially identified based on a few reference books and FishBase: 80 (belonging to 19 families) were finally included in the database; for the others, very little information was found (31). The third task consisted of searching and reading publications. It appeared particularly difficult because most relevant references were dispersed and old (usually not available online at that time, but see https://www.biodiversitylibrary.org/). This required going to different public institutions, such as the National Museum of Natural History in Paris, which host old articles, and manually photocopying hundreds of articles or other references. The fourth task consisted of manually entering data into an Excel® sheet, which was performed by the last author and lasted for ∼1.5 years. After ∼2 years of work, >80% of the 4000 cells (80 species × 50 traits) of the database were filled corresponding to the analysis of >1000 references (31).

**Table 1.** Life history trait definitions in STOREFISH [from Teletchea et al. (31)]

Life stage	Trait Nb	Trait	Primary data	Type	Secondary data	Annotation
Egg	1	Oocyte diameter (mm)	Average diameter of the unfertilized egg, i.e. mature, fully yolked ovarian oocyte ready to be or just released (in mm)	Numerical	One value	regexp
Egg	2	Egg size after water-hardening (mm)	Average diameter of the fertilized egg after swelling, due to water uptake (in mm)	Numerical	One value	regexp
Egg	3	Egg buoyancy	Egg buoyancy of the fertilized swollen egg, being either demersal (sinks to the bottom), semi-pelagic (remains for a short period in the water column, then sinks) or pelagic (floats on or near the surface of the water and could derive for a long distance, several kilometres), scored as a three-state variable [demersal, semi-pelagic and pelagic]	Categorical	Demersal Semi-pelagic Pelagic	regexp + human curation
Egg	4	Egg adhesiveness	Egg adhesiveness of the fertilized egg after water-hardening: eggs, which are slightly sticky only prior to water-hardening, were considered as non-adhesive, scored as a binary variable [non-adhesive, adhesive]	Categorical	Adhesive Non-adhesive	regexp + human curation
Egg	5	Incubation time (days)	Average number of days required in natural conditions from fertilization to hatching, period designed as ‘incubation’	Numerical	One value	regexp
Egg	6	Temperature for incubation (°C)	Average temperature, in °C, encountered during the incubation period of egg	Numerical	One value	regexp
Egg	7	Degree-days for incubation (°C/day)	Average degree-days (temperature × time, with temperature in °C and time in days), required for the entire incubation of egg	Numerical	One Value	regexp
Larvae	8	Initial larval size	Average larval size upon hatching (in mm)	Numerical	One value	regexp
Larvae	9	Larvae behaviour	Behaviour of the larvae prior to exogenous feeding, being either demersal (remain near the bottom) or pelagic (swim actively near the surface), scored as a binary variable [demersal, pelagic]	Categorical	Demersal Pelagic	Human curation
Larvae	10	Reaction to light	Larvae during the first days after hatching are either negatively (photophobic) or positively (photopositive) attracted to light, scored as a binary variable [photophobic, photopositive]	Categorical	Photophobic Photopositive	Human curation
Larvae	11	Temperature during larval development	Average temperature, in °C, encountered until the post-larvae stage (i.e. while starting exogenous feeding)	Numerical	One value	regexp
Larvae	12	Sibling intracohort cannibalism	Intra-specific predation of members of the same cohort during the post-larval growth, scored as a binary variable [absent, present]	Categorical	Absent Present	regexp
Larvae	13	Full yolk-sac resorption	Average period of time, in degree-days, required for the complete resorption of the yolk-sac	Numerical	One value	Human curation
Larvae	14	Onset of exogeneous feeding	Average period of time, in degree-days, required for the beginning of the exogenous feeding	Numerical	One value	Human curation
Life Stage	Trait Nb	Trait	Primary data	Type	Secondary data	Annotation
Female	15	Age at sexual maturity	Average age at maturity (in years)	Numerical	One value	regexp
Female	16	Length at sexual maturity	Average total body length at maturity (in cm)	Numerical	One value	regexp
Female	17	Weight at sexual maturity	Average body weight at maturity (in kg)	Numerical	One value	regexp
Female	18	Female sexual dimorphism	Females of some species develop secondary sexual characters during the breeding season, scored as a binary variable [absent, present]	Categorical	Absent Present	Human curation
Female	19	Relative fecundity	Average number of eggs (in thousands) per kilogram of body weight	Numerical	One value	regexp
Female	20	Absolute fecundity	Average number of eggs (in thousands) recorded by individual female	Numerical	One value	regexp
Female	21	Oocyte development	Synchronous (all oocytes present within the ovary are at the same stage of development), group-synchronous (at least two distinct populations of oocytes at different development stages) and asynchronous (oocytes at all stages of development), scored as a three-state variable **[synchronous, group-synchronous, asynchronous]**	Categorical	Synchronous Group-synchronous Asynchronous	Human curation
Female	22	Onset of oogenesis	Defined as the months in the year when an initial significant inflexion and increase in the GSI is observed. This period corresponds to the onset of the active vitellogenesis, mainly endogenous	Categorical	One month	Human curation
Female	23	Intensifying oogenesis activity	This is defined as the months in the year when the GSI displays the largest increase. This period mainly corresponds to the end of the active vitellogenesis, prior to the final maturation of oocytes (i.e. oocyte meiotic resumption and ovulation)	Categorical	One month	Human curation
Female	24	Maximum GSI value	This corresponds to the average maximal GSI value (%) observed within the entire reproductive cycle	Numerical	One value	regexp
Female	25	Oogenenesis duration	This corresponds to the total duration of the oogenesis process from the initial significant inflexion and increase in GSI until ovulation (in months)	Numerical	One value	regexp
Female	26	Resting period	This corresponds to the duration of gonadal quiescence between two consecutive reproductive cycles when the ovaries are recovering from the spawning act (in months)	Numerical	One value	regexp
Male	27	Age at sexual maturity	Average age at maturity (in years)	Numerical	One value	regexp
Male	28	Length at sexual maturity	Average total body length at maturity (in cm)	Numerical	One value	regexp
Male	29	Weight at sexual maturity	Average body weight at maturity (in kg)	Numerical	One value	regexp
Male	30	Male sexual dimorphism	Males belonging to certain species develop secondary sexual characters during the breeding season, scored as a binary variable [absent, present]	Categorical	Absent Present	regexp
Male	31	Onset of spermatogenesis	This is defined as the months in the year when a significant increase in the GSI is observed. This period mainly corresponds to the initial proliferation of spermatogonia through repeated mitotic divisions, and primary spermatocytes differentiation	Categorical	One month	Human curation
Male	32	Main spermatogenesis activity	This is defined as the months in the year when the GSI displays a sharp increase. This period mainly corresponds to the transformation of spermatocytes into mature spermatozoa (including the spermiogenesis process) and prior to spermiation	Categorical	One month	Human curatin
Male	33	Maximum GSI value	This corresponds to the average maximal GSI value (%) observed within the entire reproductive cycle	Numerical	One value	regexp
Male	34	Spermatogenesis duration	This corresponds to the total duration of the spermatogenesis process from the initial proliferation of spermatogonia to spermiation (in months)	Numerical	One value	regexp
Male	35	Resting period	This corresponds to the duration of gonadal quiescence between two consecutive reproductive cycles when the testis are recovering from the spawning act (in months)	Numerical	One value	Human curation
Life Stage	Trait Nb	Trait	Primary data	Type	Secondary data	Annotation
Spawning conditions	36	Spawning migration distance	Average distance run by adults to get to the spawning grounds (in km)	Numerical	One value	regexp
Spawning conditions	37	Spawning migration period	Months in the year when some species display extensive spawning run	Categorical	Months	Human curation
Spawning conditions	38	Homing	Accurate returning behaviour of some teleosts to their natal areas to spawn, scored as a binary variable [absent, present]	Categorical	Absent Present	Human curation
Spawning conditions	39	Spawning season	Usual months of the presence of spawners on the spawning ground	Categorical	January–March April–May June–July August–September October–December	Human curation
Spawning conditions	40	Spawning period duration	Period of time when spawners are present on the spawning ground (in weeks)	Numerical	One value	regexp
Spawning conditions	41	Spawning temperature	Average temperature observed during the spawning period (°C)	Numerical	One value	regexp
Spawning conditions	42	Spawning water type	Kind of water frequented during the spawning season, could be either stagnant water such as ponds or lakes (with no or slight current) or rivers and streams (with much more current), scored as a binary character [stagnant water, flowing or turbulent water]	Categorical	Stagnant water Flowing or turbulent water	Human curation
Spawning conditions	43	Spawning depth	Average depth at which spawning occurs (in m)	Numerical	One value	regexp
Spawning conditions	44	Spawning substrate	Teleost species either scatter their eggs in the water column (pelagophils), or deposit their eggs (i) on a rock or gravel bottom (lithophils), (ii) on plants (phytophils), (iii) on roots or grass above the sandy bottom or on the sand itself (psammophils) or (iv) into gill cavity of mussels (ostracophils). This character is mainly derived from Balon (1975) classification of reproductive guilds of teleost fishes, and scored as a five-state variable [pelagophils, lithophils, phytophils, psammophils, ostracophils]	Categorical	Pelagophils Lithophils Phytophils Psammophils Ostracophils	Human curation
Spawning conditions	45	Spawning site preparation	Teleost species scatter their eggs either in the water column, directly over the substrates, or within a nest, which is a depression dug into the substrate by either the male, the female or both parents, scored as a five-state variable [open water/substratum scatter, substrate chooser, nest built by male, nest built by female, nest built by both parents]	Categorical	Open water/substratum scatter Susbtrate chooser Nest built by male Best build by female Nest built by both parents	Human curation
Spawning conditions	46	Nyctemeral period of oviposition	Main period during the day when mass spawning occurs, scored as a four-state variable [night, dawn, day, dusk]	Categorical	Night Dawn Day Dusk	Human curation
Spawning conditions	47	Mating system	Teleost species display three main kind of mating system: monogamous (one male and one female), polygamous (an individual, either the male or the female, has several mates), and promiscuity (both sexes have multiple partners within a single season), scored as a four-state variable [monogamy, polygyny, polyandry, promiscuity]	Categorical	Monogamy Polygyny Polyandry Promiscuity	Human curation
Spawning conditions	48	Spawning release	Teleost species display three main kind of egg release during the breeding season: total (all eggs are shed at the same time), fractional (several batches of eggs are released at intervals, usually over several days or weeks, but the potential breeding season fecundity is fixed before spawning, also known as determinate fecundity) or multiple (several batches of eggs are shed more than once through a long spawning season, and there is a recruitment to the stock of spawnable oocytes during the entire spawning season, also known as underminate fecundity), scored as three-state variable [total, fractional, multiple]	Categorical	Total Fractional Mutliple	Human curation
Spawning conditions	49	Parity	Teleost species are either iteroparous (most individuals survive after the spawning act, i.e. several reproductive cycles during a lifetime) or semelparous (most or all individuals die, i.e. only one reproductive cycle during a lifetime), scored as a binary variable [semelparous, iteroparous]	Categorical	Semelparous Iteroparous	Human curation
Spawning conditions	50	Parental care	Association between one or both parents and offspring that enhances offspring development and survival (e.g. males of some species guard and aerate their eggs and larvae for several weeks), scored as a four-state variable [no care, male parental care, female parental care, biparental care]	Categorical	No care Male parental care Female parental care Biparental care	Human curation

Each trait was characterized as number, text, or hybrid according to the comment indicated in the initial version of the database. The Annotation column indicates the method used to transform data comments.

From these original or primary data, a set of secondary data (16) was manually generated by the last author for species (65 out of 80) and traits (29 out of 50) for which enough information was available. Based on this new data set, a first study aimed at establishing a typology of reproductive strategies to evaluate whether it is possible to extrapolate the knowledge acquired on one species to other (37); this new classification confirmed that extrapolations concerning biological traits cannot be based on phylogeny only and differed significantly from classifications earlier proposed (8, 9, 13, 14). Two additional studies (i) demonstrated that the relationship of oocyte diameter and temperature to incubation time for temperate freshwater is different from marine fish (38) and (ii) highlighted how the different trade-offs at the early life stages ensured that first feeding of larvae of temperate freshwater fish occurs in spring and early summer (39). A fourth study summarized the differences in reproductive traits between freshwater and marine fish and highlighted the possible implications for aquaculture practices (40). Altogether, the five articles linked to the STOREFISH project were cited by ∼200 articles (including 47 self-citations), with a mean of 14 ± 8 per year. The articles are grouped into five categories: basic biology (*n* = 80), aquaculture (*n* = 61), climate change (*n* = 36), invasive species (*n* = 9) and fisheries management (*n* = 7). This assessment demonstrates that the potential applications of this project, as expected in 2007, go well beyond aquaculture and that many researchers are looking for open-access data (12, 16). In addition, three of the four articles using the data in STOREFISH were much more cited than the original publication, which partly explains the reluctance of data holders to make data available because of the lack of proper citation (16). Also, even though we applied for several grants in the past decade to develop an online version and enlarge the database, we were never able to obtain any funding (35). In conclusion, the STOREFISH project may illustrate why data holders are reluctant to make primary data available given the relatively limited perceived advantages, the effort involved in preparing the data as well as the lack of funding and proper citation; so altogether, the lack of databasing work by research institutions (16, 41).

The aim of the present article is to describe STOREFISH 2.0, an online database easing the sharing of the original data published in 2007 (31), enriched with automated data extraction and presentation to display the knowledge acquired on temperate freshwater fish species. It is anticipated that this new database could be useful for freshwater biodiversity research, conservation, assessment and management (12, 16).

## Material and methods

### Data processing

Each trait was isolated from the original Excel® database and modelled to establish how secondary data should be automatically generated (Table 1). The relevant columns and lines were first exported as a csv file and then processed using Python regular expression. The general overview of the process used to extract primary data is presented in Figure 1. For categorical traits where one or more categories were present, a keyword search was performed on the primary data. If the keyword(s) search was successful, the category value was assigned, otherwise an expert curation was required to avoid any ambiguity. For numerical traits, three type of values were mostly present: (i) one number, which was extracted as is; (ii) one interval if a hyphen was between two numbers—in this case, the mean of the two numbers was calculated; and (iii) multiple numbers (single or interval)—in this situation, only the first value (or mean of an interval) was stored. Some traits contained only qualitative data as in egg buoyancy, or spawning substrate and other traits contained both numerical and qualitative values as egg diameter or larval size upon hatching (31). In this case, both numerical and categorical extractions may be performed but only the relevant data type is displayed in STOREFISH 2.0 (Table 1).

### Database setup

We used the Django framework from previous database setup such as a in the repository of red blood cell proteins called RESPIRE (42) and a web portal for virtual screening management called dockNmine (43). We reused some of the core routines in STOREFISH 2.0, for instance those involving mining routines making use of Biopython (44) and those for literature management. The database is powered by Apache 2.4 web server running on a virtual machine powered by Ubuntu 18.04 LTS, mysql 5.6 is used to store data content. To enhance the user experience, bootstrap 3.0 and Amcharts javascript libraries are used. The database content is backed up every day.

## Results and discussion

### Transformation of primary data into secondary data

The original STOREFISH database was filled in with 3256 unique traits description, i.e. 81.4% out of a maximum of 4000 annotations (80 species with 50 traits each). These traits listed in 1949 Excel® lines were first split into 14 836 primary data in csv files as many lines contained more than one unique annotation. The processing of these csv files allowed us to identify 8236 primary data for numerical traits and 6600 primary data for categorical traits. The processing of primary data using regular expressions for numerical traits allowed to recognize 1836 single numerical values, to extract the mean of 2518 numerical intervals, and 3396 additional numbers when more than one number was found in the original annotation. This secondary data processing allowed to extract 94% of the primary data found in the initial version of STOREFISH for numerical traits. The unambiguous assignation of traits by category could also be performed for 53% of primary data. The remaining ambiguous cases required expert curation; some primary data were not conclusive for the considered trait. A few examples for each of the treatments and special cases requiring human curation are indicated below.

Depending on the difficulty of treatment of the primary data content, three types of processing routines were performed (Table 2). In the first case, computational transformation allowed to unambiguously determine the secondary data value. For oocyte diameter in *Barbatula barbatula*, a single numerical value ‘1’ was present in the primary data; therefore, the number was recognized ‘as is’ without any further treatment and converted into a float numerical value of 1.0. When an interval was found, such as for the oocyte diameter of *Tinca tinca* (0.4–0.5), it was converted into the average float value of 0.45. When more than one number was detected, as for the oocyte diameter in *Lepomis gibbosus* (0.529 and 0.477), only the first one was kept. Primary data may also contain mean values with the plus–minus separator sign. In that situation, the mean value was kept and the standard deviation was not considered. In all three situations, only a single numerical value was extracted, converted when possible to the mean value for the parameter. When no numerical value was present, like in the oocyte diameter for *Aphanius iberius* (‘big eggs’), no secondary data was obviously computed. Data processing was equally unambiguous for categorical data where a single keyword, or a short list of keywords, defines the trait. The ‘photophobic’ status for the reaction to light trait in *Alosa alosa* was straighforward from the single value found in the primary data, as for the spawning season (months) for *Oncorhynchus tshawytscha* (September and October). Even when complex sentences were present in the primary data, as in the sibling intracohort cannibalism annotation for *Esox lucius*, exact keyword matching (‘cannibalism’) allowed to qualify the category without error. In this long sentence, multiple data were present: the expert indicated that the starting day of cannibalism was present, alongside the fish length at this time. Those traits are however not addressed in the present database, but we plan to extract more secondary data in the future.

**Table 2.** Examples of transformation of primary data into secondary data.

Trait(db id)	Trait type	Primary data	Processing type	Secondary data	Expert curation
**Unambiguous data assignation**					
Oocyte diameter (9054)	Numerical	1 mm	Regexp (single value)	1 mm	Not needed
Oocyte diameter (9223)	Numerical	0.4–0.5 mm	Regexp (interval)	0.45 mm	Not needed
Oocyte diameter (9293)	Numerical	The mean diameter of ripe eggs in ovaries of females in Upper Beverley Lake was 0.529 mm, signigficantly larger than 0.477 mm in Lower Beverley Lake	Regexp (first value)	0.529 mm	*Not needed, location is not taken into account*
Oocyte diameter (9054)	Numerical	5.186 ± 0.263 mm for oocytes at the final maturation stage [For broodstock population cultured in a fish farm in Southern Chile]	Regexp (mean)	5.186 mm	Not needed
Oocyte diameter (9046)	Numerical	Big eggs	No data found	No data	**No value detected, valid secondary data**
Reaction to light (11 339)	Categorical	Photophobic	Keyword	Photophobic	Not needed
Spawning season (10 792)	Categorical	From late September to early October	Keyword	[‘September’, ‘October’]	Not needed
Reaction to light (11 339)	Categorical	Photophobic	Keyword	Photophobic	Not needed
Sibling intracohort cannibalism (11 587)	Categorical	At 28–35 days, cannibalism occured indepedently in all 12 tanks. The mean age at first cannibalism was 32 days (SD = 1.5 days) which occured at a mean length of 30.3 mm (SD = 4.3 mm)	Keywords	Present	**Valid annotation, but more information could be extracted**
**Ambiguous data assignation**					
Onset of oogenesis (7857)	Categorical	A slight increase from November until March	Keywords	[‘March’, ‘November’]	**Need to be updated to taken into account intermediate months**
**Errors or uncertainties**					
*Oocyte diameter (9072)*	*numerical*	*In 70–80% of the females, two distinct generations of egg cells were observed before Spawing: oocyte diamters in the range 0.822–0.946 and 0.316–0.550 mm, respectively*	*Regexp (first value)*	***70***	**Required, should be one mean of the two intervals, not 75%**
Oocyte diameter (9331)	Numerical	Mean ooocyte diameter from all females at the second sampling (20 March) was 838 ± 18 µm. When oocytes completed FOM and were ovulated (1131 ± 20 µm in diameter)	Regexp (mean)	838 µm	**Value is stored in µm but should be in mm, correct value is 0.838 mm**

In the second case, transformation required more attention than single numerical or categorical value extraction detailed earlier. The values extracted were correct but incomplete. An example is given for the Onset of Oogenesis in *Ictalurus punctatus* where 2 months were correctly detected (November and March), but the intermediate months were not taken into account. Indeed, primary data contained the period description (from November until March), but many literal forms may exist even in this simple case: (i) from November to March, (ii) starting in November and ending in March and (iii) ending in March after slow increase in November. Since all sentences may be valid English (or non-native writing in English) and can be found in publications, caution must be taken and expert curation was performed to adjust the initial list to a more correct one (November, December, January, February and March). In most cases in this situation, no secondary data were extracted; so, the expert curation is mandatory to validate data, if they should be present; such primary data have never been analysed before (31, 37–40) and explained why they required additional work to be transformed into reliable secondary data.

In the third case, the aforementioned methods lead to inconsistencies or errors. Expert curation was then mandatory to correct these errors. We illustrate these situations with two examples for the oocyte diameter trait. For the species *Blicca bjoerkna*, three interval values were detected. Due to the sentence ordering, a percentage was wrongly transformed into millimetres, the secondary data becoming 75.0 mm instead of 0.884 mm (mean of the valid first interval). Since there were three intervals (and thus six numerical values), only an expert can indicate which value has to be kept in the secondary data annotation. After curation, the secondary data extraction was kept, but the curated and corrected value was saved in a dedicated field in the database. A more complex example is for *Morone saxatilis*. For this species, the numerical value picked was the correct one (838), but the authors had indicated the numerical value in micrometres instead of millimetres, as found in most articles. This situation could be handled using complex bioinformatics treatments, but after evaluation, we found the expert annotation to be more robust, in particular less prone to false-positive detection.

**Figure 2.**
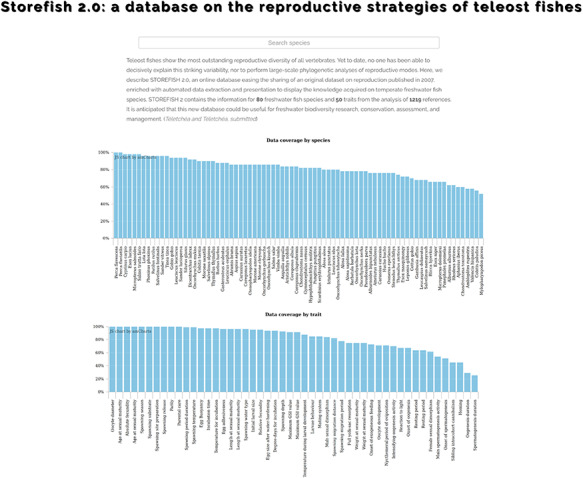
STOREFISH home page. Accessible data are available by species, traits or by reference. A search-as-you-type box allows to rapidly find a species of interest.

After careful analysis of the primary data present in the initial STOREFISH, we have set up a limited set of transformations to ensure that most data were transformed into numerical and categorical data unambiguously. These transformations were kept conservative, with simple transformation rules, so our expertise could concentrate on the most tedious cases, where multiple data were detected (up to 36 numerical in primary data for some entries). The programmatic transformation of data led to the automatic annotation of 75% of the original corpus, manual curation added an extra 10% and the remaning values will need further evaluation (contradictory or non-existing data in primary data) for a future release of the database. Upon curation, we have set up more rigid naming conventions for future data incorporation and identified the need to have shorter primary data text extraction, which could ease the arrival of new curators.

**Figure 3.**
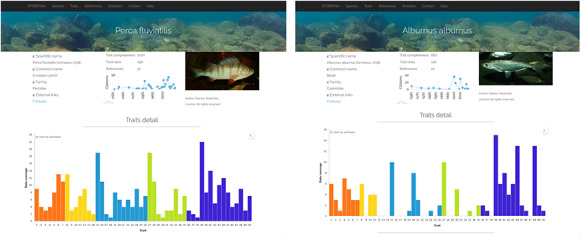
Comparison of trait enrichment between *Perca fluviatilis* (442 primary data, resulting in 237 secondary data) and *Alburnus alburnus* (146 primary data, resulting in 102 secondary data).

### Overview of STOREFISH 2.0

This database contains the information for 80 freshwater fish species and 50 traits from the analysis of 1219 references (Figure 2). After a short description of the content of the database, the first page presents two graphs displaying the data coverage by species and traits, both classified from the best to the least known, which allows a rapid assessment of the knowledge acquired in the past decades (Figure 2). The data coverage ranges from 100% for yellow perch *Perca flavescens* to 52% for black carp *Mylopharyngodon piceus*, and from 100% for oocyte diameter to 25% for spermatogenesis duration (Figure 2). The second page presents the 80 species with their scientific name, order, family and common name. By default, species are alphabetically ordered, but they can also by classified by their order, family or common name. Species can be displayed one by one with on the top of the page an overview of the trait completeness, total data found and number of references used; and when available, a picture provided by the last author (45, 46). An external link to FishBase was also added. Then, a graph shows the number of data found for each of the 50 traits with a different colour for egg, larvae, female, male and spawning conditions. Two contrasting examples are provided in Figure 3, which highlights a species, European perch *Perca fluviatilis*, for which many information has been found and one barely known, bleak *Alburnus alburnus*. These two examples illustrate that most studies focused on economically valuable species and conversely fish species with a small distribution range as well as endemic species present lower coverage and data availability in trait databases (12). This represents the biggest obstacle for biodiversity data users because many species and regions are still highly under-sampled or completely unrepresented (e.g. rare taxa, regions that are difficult to access) in online databases (10, 25). Conversely, it also demonstrates that we accumulated considerable traits information for some species by painstakingly reviewing accessible literature (11). Then, five tables present in detail the information for each trait, with five columns: trait id, trait, primary data (raw information as originally entered in the Excel® sheet by the last author), secondary data (extracted as explained in Figure 1) and reference. We chose to provide primary data online because it allows detecting knowledge gaps regarding trait information and possible discrepancies among fish-trait databases (12). It could also be useful to evaluate the intraspecific variability of traits, that is, differences of traits within species for instance due to different environments or geographical variation (12). Intraspecific trait variability is frequently neglected as trait values are summarized as averages per species; thus, only secondary data (16) are available in most publications, or it is assumed to be negligible compared to interspecific variability (37–39), which might lead to biased results (10, 12). Secondary data were automatically extracted and manually curated when necessary (Figure 1), and then used to perform the graphs as well as the univariate statistics showed in the database.

**Figure 4.**
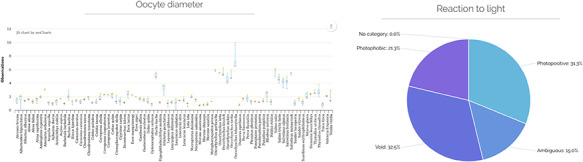
Example of detailed database statistics for numerical traits (oocyte diameter, left) and categorical traits (reaction to light, right). The graphs are interactive online, discrete values are shown upon mouse hovering on the displayed data. Void categories indicate there is no primary data, No category indicates that no category was detected, ambiguous values indicate that multiple categories were found for one species, with no category being more representative of the secondary than any other.

The third page presents for each of the 50 traits, grouped into the five main categories, the definition described in Teletchea *et al.* (31). It is possible to display all information acquired for each of the 50 traits by clicking on it. The third page presents the reference with a graph showing the number of references per year. The oldest reference was published in 1928, while the most recent analysed is in 2010; the last author stopped looking for references in the past decade. It is anticipated that many more articles have been published on the reproduction of those species and therefore new data will now be regularly added. By clicking on each reference, it is possible to access all data extracted in the database linked to it. The fourth page entitled statistics presents univariate analysis from the secondary data, using box plots for numerical traits, and pie charts for categorical traits (Figure 4).

### Future updates of STOREFISH 2.0

Although the call for open-access data is becoming louder, long-term and large-scale data are still difficult to obtain (16). This may be due to the fact that only few water managers, policymakers or even scientists are aware that globally shared open-access data can be useful (16). Developing an online database is a long journey and first requires the clear standardization of trait definitions, better known as ontologies, which are not yet available for many taxa, hampering data gathering and sharing (10, 19). Such standardization may result in researchers concentrating their efforts on a limited number of traits (50 in STOREFISH) and, therefore, reducing research of other traits that may be revealing for particular groups or poorly understood ecological functions (10). Then, standardized protocols and corresponding database tools are required for recording trait data but are not yet available or applied in freshwater fish (12). Besides, the capture of phenotypic information in natural language in a way that is amenable to computational analysis has been a major challenge of the past two decades (19). Therefore, till today, data are still entered by dedicated encoders (24). Encoding does not only imply manual typing of data; rather, it is that aspect of doing science that involves searching the literature for pertinent information, breaking this information down to units of data and finally encoding these into an interface incorporating rules and error traps, as does the FishBase encoding interface (24). In contrast to FishBase (1) or Fishtraits (11), all information in STOREFISH was entered by one person (F.T.), and no data entries were verified by a second person. Therefore, we will continue to use feedback from peer reviewers and users of the database, as well as our continuing review of literature, to update and correct it, as performed for FishTraits (11) and FishBase. As any database, a user should always check the date of last update, gain a basic understanding of the data flows and be aware of the risk of error propagation (16). Despite the multitude of possible pitfalls and limitations in the usage of data from different sources, the benefits of having them publicly available clearly outweigh the potential issues (16). Public availability of data exposes them to possible scrutiny by peers, opens the potential to reuse including integration in large-scale analyses, represents an increased resource efficiency (not requiring new investments in data generation for well-covered areas) and results in a better understanding of gaps in the data (16). To make sure that our database will remain accessible, which is a major issue due to the lack of resources to manage and preserve data for the long term [see Costello et al. (47)], we plan to make STOREFISH a fully machine-readable linked open data (16) and adhere to the FAIR principles, namely making data findable, accessible, interoperable and reusable, to be able to join the Open Traits Network (41).

## Conclusion

Nearly 15 years after the onset of the STOREFISH project, the database is ultimately online. Even though technologies have improved during this time, human curation remains essential at each step of the process, particularly for searching and encoding primary data (23, 24). Yet, the transformation of primary data into secondary data can be quite efficiently performed automatically if primary data are correctly entered within the database. Now that we know better how to enter the primary data to automatically extract the information, we anticipated to release an enlarged database next year, focusing on the early life stages of American freshwater fish species (48). In the future, our main goal is to combine the data gathered within STOREFISH 2.0 and similar databases with concept and methods of systematics (33, 34), to better understand the evolution of life history across the tree of life of fishes as well as help answering more applied questions, such as the onset of cannibalism or the link between larval size and feeding protocols (33, 34). We also anticipate that this new database could be useful for freshwater biodiversity research, conservation, assessment and management.
